# Lateral Curvature of the Spine in Children

**Published:** 1907-04-27

**Authors:** L. A. Parry


### THE GENERAL PRACTITIONERS' COLUMN.

### LATERAL CURVATURE OF THE SPINE IN CHILDREN.

By L. A. PARRY, M.B., B.S.Lond., F.R.C.S.

LATERAL curvature of the spine in children is a common affection. No doubt a large number of these cases get quite well, but in a certain number the disease steadily goes from bad to worse, and ultimately leaves the child a permanent cripple. Hence I think it cannot be out of place to call attention to the urgent importance of early, prolonged, and systematic treatment.

### HOW TO EXAMINE THE CASE.

The examination of these cases is important. The child is stripped, and the position of the spines of the vertebræ is marked by a carbon pencil. Any deviation, unless it be exceedingly slight, is thus readily detected. In cases where the amount of curvature is quite small, it is made more manifest by getting the child to lean well forwards, with its arms crossed on the chest. Any difference in level of the anterior superior spines of the ilia is next looked for, as one of the first points in treatment is to correct any obliquity of the pelvis. The child is next suspended by lifting it up under the armpits, to see if any alteration is made in the curve. This procedure is important for prognosis. If the curve is obliterated, one may reckon that proper treatment will succeed in completely restoring the spine to its normal shape, but if the curve is only diminished, or is not altered, the spine can never be restored to its correct shape; the case can at the best be cured with such an amount of permanent deformity as is due to bony change.

### POINTS IN TREATMENT.

The first point in treatment is, as in every other disease, to remove any possible cause. If the two limbs are of unequal length, giving rise to obliquity of the pelvis, this must be remedied by some suitable means, such as a proper boot, some surgical operation, etc. Any attitude which is a bad one, and is constantly assumed by the child, must be most carefully corrected. Stooping forwards to read as a result of myopia is an example of this. It is always well to see if the child has enlarged pharyngeal or faucial tonsils, and remove them if necessary.

Drugs are of importance. Anæmia, dyspepsia, and constipation all require treatment, and must immediately be seen to. General hygienic treatment is necessary. Plenty of fresh air, change of climate, and careful diet are to be ordered.

As regards ordinary exercise, great care is necessary. It is important, on the one hand, not to let the child overtire itself; and, on the other hand, a certain amount of healthy outdoor exercise is essential. All children should lie down flat, either in the prone or supine position, for at least two hours a day. One hour should for preference be immediately after the special exercises to be referred to below; the second hour at some other time. Massage for half an hour daily to the muscles of the back is an important procedure.

### IS A SUPPORT NECESSARY?

As regards spinal supports, very little need be said. Modern treatment most emphatically condemns them in all but the most exceptional cases, and these are the cases in which the curvature steadily increases in spite of careful treatment, or in which the pain or weakness of spinal muscles is very marked. I have only had one example in which any support has been necessary. In the rare cases in which a support is needed, spinal braces are probably the best. Even then the apparatus should not be worn continuously, but only at intervals during the day, and it should be of such a nature that it does not prevent movement of the spine and its muscles.

The special exercises which are to be made use of in the treatment of scoliosis are very important. They must be regularly and systematically carried out. At first the child should be taught the exercises by the surgeon himself, in the presence of the mother or governess who is going to see to them at home. They commence with easy ones, lasting only a few minutes; the time is gradually increased as the back becomes stronger, and the exercises more difficult, to half an hour twice a day. They should be carried out slowly, so as not to tire the child, with frequent intervals of rest, at first in the supine position, later standing up, and later still using light dumb-bells—at first not exceeding 1 lb. in weight. I do not propose to describe the exercises in detail; space does not permit that. I employ those recommended by Lewis, but I attach a great deal more importance to the regular, persistent, and systematic carrying out of a scheme of exercises than to any particular variety or plan. Briefly such exercises consist of a series of movements which exercise the muscles acting on every part of the body, the limbs, the head, and the trunk by flexion, extension, abduction, adduction, and circumduction. In early cases a month or six weeks will cause the curvature to disappear.

### EXERCISES.

If the curvature is very marked, special exercises must be designed. For example, in a dextro-dorsal curvature the following is an excellent one. The right hand should be placed on the ribs, as high up and as far back as possible, thumb forwards. The left forearm is placed on the head, so that the left fingers touch the right ear. Bend all the body above the right hand as far as possible to the right, and whilst in that position take a few deep inspirations, then return to the symmetrical position. This is merely an example; other exercises can be designed for other curves. I conclude by once more repeating that by immediate and proper treatment one can generally save early examples of scoliosis from passing into confirmed cripples, and that the treatment can be carried out by any ordinary practitioner if he have sufficient care and patience.

